# Pharmacokinetics in mice and growth-inhibitory properties of the putative cancer chemopreventive agent resveratrol and the synthetic analogue *trans* 3,4,5,4′-tetramethoxystilbene

**DOI:** 10.1038/sj.bjc.6601568

**Published:** 2004-02-03

**Authors:** S Sale, R D Verschoyle, D Boocock, D J L Jones, N Wilsher, K C Ruparelia, G A Potter, P B Farmer, W P Steward, A J Gescher

**Affiliations:** 1 Cancer Biomarkers and Prevention Group, Department of Oncology, University of Leicester, Leicester, UK; 2 Cancer Drug Discovery Group, School of Pharmacy, DeMontfort University, Leicester, UK

**Keywords:** chemoprevention, drug design, metabolism, resveratrol, stilbene

## Abstract

Resveratrol (*trans*-3,5,4′-trihydroxystilbene) is a naturally occurring polyphenol with cancer chemopreventive properties in preclinical models of carcinogenesis, including those of colorectal cancer. Recently, a variety of analogues of resveratrol have been synthesised and investigated in *in vitro* assays. One analogue, 3,4,5,4′-tetramethoxystilbene (DMU 212), showed preferential growth-inhibitory and proapoptotic properties in transformed cells, when compared with their untransformed counterparts. As part of a chemoprevention drug development programme, the pharmacokinetic properties of DMU 212 were compared with those of resveratrol in the plasma, liver, kidney, lung, heart, brain and small intestinal and colonic mucosa of mice. DMU 212 or resveratrol (240 mg kg^−1^) were administered intragastrically, and drug concentrations were measured by HPLC. Metabolites were characterised by cochromatography with authentic reference compounds and were identified by mass spectrometry. The ratios of area of plasma or tissue concentration *vs* time curves of resveratrol over DMU 212 (AUC_res_/AUC_DMU212_) for the plasma, liver, small intestinal and colonic mucosa were 3.5, 5, 0.1 and 0.15, respectively. Thus, resveratrol afforded significantly higher levels than DMU 212 in the plasma and liver, while DMU 212 exhibited superior availability compared to resveratrol in the small intestine and colon. Resveratrol was metabolised to its sulphate or glucuronate conjugates, while DMU 212 underwent metabolic hydroxylation or single and double *O*-demethylation. DMU 212 and resveratrol inhibited the growth of human-derived colon cancer cells HCA-7 and HT-29 *in vitro* with IC_50_ values of between 6 and 26 *μ*M. In the light of the superior levels achieved in the gastrointestinal tract after the administration of DMU 212, when compared to resveratrol, the results provide a good rationale to evaluate DMU 212 as a colorectal cancer chemopreventive agent.

Resveratrol (*trans* 3,5,4′-trihydroxystilbene, for structure see Figure 1

**Figure 1:**
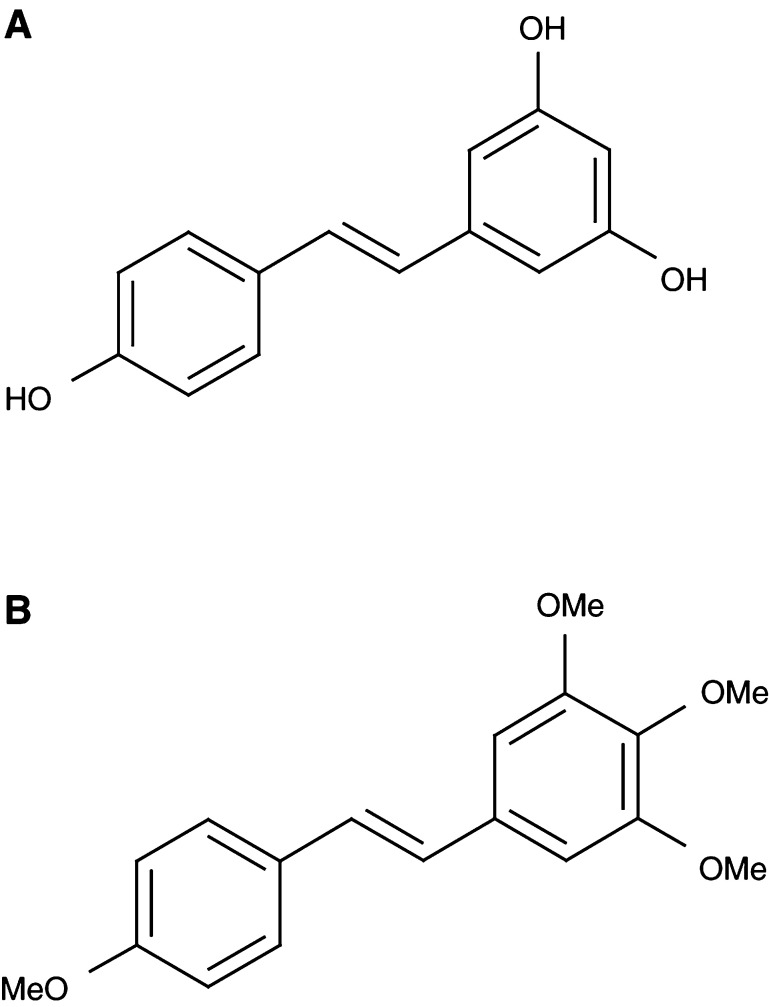
Chemical structures of (**A**) resveratrol and (**B**) DMU 212.

) is a phytoalexin generated in response to environmental stress or pathogenic attack in grapes, mulberries, cranberries, peanuts and plants of the *Cassia quinquangulata* family. Resveratrol inhibits diverse cellular events associated with the three major stages of carcinogenesis: initiation, promotion and progression (Jang *et al*, 1997). Since this discovery, resveratrol has been the subject of a large number of preclinical and mechanistic studies. The cancer chemopreventive potential of resveratrol has been demonstrated in the models of carcinogenesis *in vivo* and in cells *in vitro*. It inhibits the proliferation of a variety of cancer cell lines (for a review, see Gusman *et al*, 2001), formation of preneoplastic lesions in the 7,12-dimethylbenz(*a*)anthracene-(DMBA) induced mouse mammary organ culture model (Bhat *et al*, 2001) and benzo(*a*)pyrene-induced transformation of rat tracheal epithelial cells (Jang *et al*, 1997). In animal studies, resveratrol interfered with the formation of azoxymethane-(AOM) induced aberrant crypt foci in rat colon (Tessitore *et al*, 2000), attenuated oesophageal carcinoma formation in rats that received *N*-nitrosomethylbenzylamine (NMBA) (Li *et al*, 2002), decreased the number of adenomas in the small intestine and suppressed tumour formation in the colon of Apc^*Min*+^ mice (Schneider *et al*, 2001) and reduced mammary tumour formation in *N*-methyl-*N*-nitrosourea-(NMU) treated rats (Bhat *et al*, 2001). Resveratrol has been reported to possess a variety of anti-inflammatory, antiplatelet and both pro- and antioestrogenic effects (Bertelli *et al*, 1996; Gehm *et al*, 1997; Jang *et al*, 1997; Uenobe *et al*, 1997). It exerts a wide variety of biological effects germane to cancer chemoprevention, including the inhibition of cytochrome *P*450 enzyme expression activity (Chun *et al*, 1999; Guengerich *et al*, 2003), induction of apoptosis (Mahyar-Roemer *et al*, 2001, 2002), modulation of components of the cell cycle machinery (Schneider *et al*, 2000; Wolter *et al*, 2001), decrease in cyclooxygenase 1 (COX-1) activity and COX-2 expression (Subbaramaiah *et al*, 1998; Mutoh *et al*, 2000; Li *et al*, 2002), antioxidation (Sgambato *et al*, 2001), inhibition of activities of protein kinase C and D (Haworth and Avkiran, 2001; Slater *et al*, 2003) and decrease in the activity of transcription factors NF*κ*B and AP-1 (Surh *et al*, 2001; Banerjee *et al*, 2002). As is the case with many polyphenols with putative cancer chemopreventive properties, the systemic bioavailability of resveratrol is probably poor. This notion is borne out by studies in mice, rats and dogs, which suggest consistently that resveratrol is well absorbed but avidly glucuronidated and sulphated both in the liver and in intestinal epithelial cells (Asensi *et al*, 2002;Juan *et al*, 2002; Marier *et al*, 2002). One study in humans also hints at a poor bioavailability of resveratrol (Goldberg *et al*, 2003). In the wake of the discovery of the interesting pharmacological properties of resveratrol, the trihydroxystilbene scaffold has become the subject of imaginative synthetic manipulations by medicinal chemists with the aim of generating novel congeners of pharmacological interest and to characterise structural features, which impart activity to the molecule. These structural alterations have been aimed at the optimisation of the cytochrome *P*450 enzyme-inhibitory and antimutagenic potencies of the molecule (Chun *et al*, 2001; Kim *et al*, 2002), its antioxidant activity (Lu *et al*, 2001), its apoptosis-inducing and growth-inhibitory activity (Lu *et al*, 2001; Nam *et al*, 2001; Kim *et al*, 2002) and its ability to inhibit cell transformation (She *et al*, 2003). These chemical synthetic attempts have predominantly been concerned with the introduction of additional hydroxy moieties into the trihydroxystilbene framework and with various degrees of methylation of the phenol groups. An especially auspicious finding concerning these analogues is the fact that 3,4,5,4′-tetrahydroxystilbene, resveratrol with an additional hydroxy moiety, and its *O*-methylated congener, 3,4,5,4′-tetramethoxystilbene (DMU 212, for structure see Figure 1), were capable of preferentially interfering with the proliferation and survival of transformed human lung-derived cells, with much lower growth-inhibitory and apoptotic properties in their untransformed counterparts (Lu *et al*, 2001). In contrast, resveratrol did not possess this discriminatory potential. Furthermore, DMU 212 is currently under preclinical evaluation as a potential antitumour prodrug that undergoes metabolic activation by certain cytochrome *P*450 enzymes (Potter *et al*, 2002a). In the light of the availability of pharmacologically interesting stilbene analogues, it seems appropriate to compare the cancer chemopreventive potential of resveratrol in preclinical models with those of its congeners, which have been shown to possess increased potency in relevant *in vitro* assays. Before embarking on such efficacy studies, it is desirable to find out if resveratrol congeners, to be evaluated as potential chemopreventive agents, possess adequate bioavailability in the tissues in which malignancies are to be prevented. Such pharmacokinetic exploration should be an essential part of the chemopreventive drug discovery process. Mindful of these considerations, we chose DMU 212, one of the most interesting resveratrol analogues described thus far (Lu *et al*, 2001; Potter *et al*, 2002a), and compared its levels in murine tissues after oral administration with those of resveratrol. Thus, the hypothesis was tested that a replacement of the phenol functionalities in resveratrol by methoxy moieties and an addition of a further methoxy group impinge on the pharmacokinetic properties of the parent molecule. Additionally, DMU 212 was compared with resveratrol in terms of their metabolism in the mouse *in vivo* and in liver homogenate preparations *in vitro*. The mouse is the animal species frequently used in cancer chemoprevention efficacy studies. Finally, we compared the ability of DMU 212 and resveratrol to interfere with the growth of human-derived colon cancer cells. Overall, the study was designed to help rationalise the choice of resveratrol analogues for further testing for potential usefulness as cancer chemopreventive agents.

## MATERIALS AND METHODS

### Reagents, animals and cells

**Figure 2:**
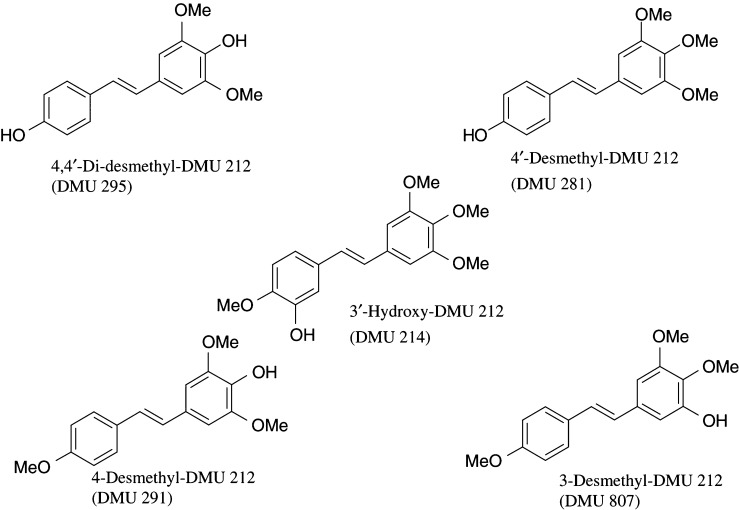
Structures of five putative metabolites of DMU 212.

Resveratrol, DMU 212 and its congeners referred to in this work are exclusively the *trans*-isomers. Resveratrol was purchased from Changchun Kingherb International Co., Ltd (Changchun, China) and its purity established as 99% by HPLC analysis. Authentic resveratrol-3-sulphate was a gift from Dr Tristan Booth (Mount Royal Pharma, Montreal, Canada), and its identity was corroborated by mass spectrometry. DMU 212 (3,4,5,4′-tetramethoxystilbene) was synthesised by Wittig olefination involving the reaction of 4-methoxybenzyl-tri-phosphonium chloride with 3,4,5-trimethoxybenzaldehyde (Potter *et al*, 1999). This reaction yielded the *-cis* and *-trans* geometric isomers, which were separated by preparative column chromatography. The *-trans* isomer was purified by recrystallisation from ethanol. The DMU 212 analogues to be used for metabolite identification of 4,4′-dihydroxy-3,5-dimethoxystilbene (4,4′-di-desmethyl-DMU 212, DMU 295), 4′-hydroxy-3,4,5-trimethoxystilbene (4′-desmethyl-DMU 212, DMU 281), 4-hydroxy-3,5,4′-trimethoxystilbene (4-desmethyl-DMU 212, DMU 291) and 3-hydroxy-4,5,4′-trimethoxystilbene (3-desmethyl-DMU 212, DMU 807) (for structures, see Figure 2

) were synthesised in a similar fashion using the *tert*-butylmethylsilyl-protected Wittig precursors, and final deprotection with tetrabutylammonium fluoride (Potter *et al*, 1999). 3′-Hydroxy-3,4,5,4′-tetramethoxystilbene (3′-hydroxy-DMU 212, DMU 214, *trans* isomer of combretastatin A4) was synthesised according to the method of Pettit *et al* (1995). The identity of newly synthesised compounds was confirmed by mass spectrometry, nuclear magnetic resonance spectrometry and infrared and ultraviolet spectroscopy. Purity was established as at least 99% by HPLC analysis. The stability of resveratrol in solution is affected by light. Therefore, care was taken to protect the solutions of compounds from light. Laboratory chemicals were purchased from Sigma Chem. Comp. (Poole, UK). Male C57BL/6J mice were obtained from Charles River Laboratories (Margate, UK). Mice were chosen for this study, as they frequently are the experimental model of carcinogenesis used in preclinical chemoprevention studies. Human-derived malignant colorectal carcinoma cell lines HT-29 and HCA-7 were obtained from Prof C Paraskeva (Bristol University, Bristol, UK) and Dr S Kirkland (Hammersmith Hospital, Imperial College, London, UK), respectively. Cells used in the experiments had been subcultured 20–30 times.

### Treatments

Animal experiments were conducted as stipulated by Project Licence 40/2496 granted by the UK Home Office. Experiments were vetted and approved by the Leicester University Animal Welfare Committee and complied with the UKCCCR guidelines for the welfare of animals in experimental neoplasia. Mice aged 8 weeks (20–22 g) received resveratrol or DMU 212 (240 mg kg^−1^ body weight, equivalent to 1 mmol kg^−1^ resveratrol or 0.8 mmol kg^−1^ DMU 212) via the intragastric route (three animals per time point). The vehicle was glycerol formal, and the dose volume approximately 10 ml kg^−1^. In the case of DMU 212, the vehicle also contained 10% DMSO. Mice were killed by terminal gaseous anaesthesia at 10, 30, 60 or 120 min *post*dosing. Blood was collected by cardiac puncture, and plasma was obtained by centrifugation. The liver, kidney, lung, heart, brain and gut were excised, and scrapings were obtained from small intestine and colon. Tissues were snap-frozen (liquid nitrogen) and stored at −80°C until analysis.

### Incubation with liver microsomes

Microsomes were prepared by differential centrifugation of mouse liver homogenate first at 9 × 10^3^ **g** (20 min, 4°C), then at 10^5^ **g** (1 h) in a Beckman L-8-60 ultracentrifuge (Beckman Coulter UK Ltd, High Wycombe, Buckinghamshire, UK). The microsomal pellet was suspended in Tris buffer (50 mM, pH 7.4), recentrifuged at 10^5^ **g** (1 h) and resuspended in Tris buffer. Liver microsomes from two to four mice were pooled. Microsomes (0.5 mg protein ml^−1^) were incubated at 37°C with NADPH (1 mM), MgCl_2_ (1 mM) and resveratrol or DMU 212 (1 mM) for 20 min (final volume: 0.2 ml). The addition of one volume of ice-cold methanol terminated the reaction. The mixture was vortexed (30 s), centrifuged (3 min, 13 400 **g**) and the supernatant was collected and analysed by HPLC. For the biosynthesis of resveratrol glucuronide for use as a reference compound, microsomes were incubated with resveratrol (1 mM) as described above, except that NADPH was replaced by uridine-diphosphoglucuronic acid (1 mM).

### Extraction of agents from plasma and tissues

Tissues were homogenised (1 : 1 volume to tissue mass ratio) in 50 mM Hepes buffer (Sigma, Poole, UK) using a hand-held glass homogeniser. An aliquot (250 *μ*l) of homogenate, to which an internal standard had been added was vortexed, followed by the addition of acetonitrile (1 ml). After vigorous shaking, mixtures were kept on ice (5–10 min) and centrifuged (2800 **g**, 10 min, 4°C). The supernatants were dried under a stream of nitrogen and reconstituted in mobile phase (100 *μ*l).

### HPLC analysis

HPLC analysis was performed using a Varian Prostar HPLC system (Varian, UK) with a Pro-Star 230 solvent delivery system, a Pro-Star 310 UV–Vis detector, a Pro-Star 363 Fluorescence detector, a 410 Varian autosampler and an Ultracarb C_18_ column (4.6 mm × 250 mm, 5 *μ*m, Phenomenex, UK). The mobile phase consisted of the three components: aqueous ammonium acetate (pH 6.5, 50 mM), propan-2-ol and acetonitrile. The gradient system that determined the composition of the eluent concerning these three components was as follows: for resveratrol 80 : 10 : 10 at the start, 75 : 10 : 15 at 10 min, 70 : 10 : 20 at 15 min, 60 : 10 : 30 at 17 min, 50 : 10 : 40 at 20 min and 20 : 10 : 70 at 25 min; for DMU 212 45 : 10 : 45 at the start for up to 10 min, 20 : 10 : 70 at 15 min and 10 : 10 : 80 at 20 min. The flow rate for both methods was 1 ml min^−1^.

Internal standards were carbamazepine and 4′-methoxy-4-methyl-*trans*-stilbene for resveratrol and DMU 212, respectively. Resveratrol and its metabolites was analysed using UV detection (325 nm), and DMU 212 and its metabolites were determined with fluorescence detection (335 nm excitation, 395 nm emission). The injection volume of samples reconstituted in the mobile phase was 50 *μ*l. The preliminary characterisation of metabolites was achieved by cochromatography using either synthetic standards or biosynthetically generated resveratrol glucuronide.

Quantification of resveratrol and DMU 212 was performed using standard curves constructed with relevant drug concentrations. The curves were characterised by regression coefficients of *R*^2^=0.99 or above. The extraction efficiencies (in percent) for resveratrol and DMU 212 were as follows: from plasma 102±17 and 67±2, respectively, from tissues 86±7 and 78±15, respectively (mean±s.d., *n*=5–7). The recovery of the resveratrol conjugates was approximately 50% that of the DMU 212 metabolites 60%. Therefore, metabolites were not quantitated.

The area under the plasma or tissue concentration *vs* time curve (AUC) values were calculated using the trapezoidal rule (WinNonlin v.1.1, Scientific Consultants, USA).

### Mass spectrometry

Mass spectrometry was performed using a Quattro Bio-Q tandem quadrupole mass spectrometer upgraded to Quattro MK II specifications (Micromass, Manchester, UK) with a pneumatically assisted electrospray interface. Samples were analysed in positive ion mode. The temperature was maintained at 120°C, and the operating voltage of the electrospray capillary was 3.88 kV and the cone voltage was 32 V. HPLC conditions used for the on-line HPLC-mass spectrometric analyses were as described for DMU 212 above.

### Effect of agents on growth of HT-29 and HCA-7 cells

Cells were seeded (10^4^ per well) in 24-well plates and cultured in DMEM containing Glutamax I (Life Technologies, Paisley, UK) and glucose (4.5 g l^−1^) and 10% foetal calf serum (Gibco, Paisley, UK). Resveratrol or DMU 212 (1–100 *μ*M) dissolved in DMSO was added to cellular incubates 24 h postplating. Cells were counted 72, 96, 120, 144 and 168 h *post*addition of agents using a Z2 Coulter Particle Count and Size Analyzer (Beckman Coulter, High Wycombe, UK). Control cultures contained the vehicle only. The amount of DMSO added to the incubate did not exceed 0.1%, which on its own failed to affect the cell growth. The IC_50_ values inserted in Figure 5 were calculated from the linear portion of the cell number-*vs* agent concentration curves at the 168 h time point.

## RESULTS

### Plasma and tissue levels of resveratrol and DMU 212

**Figure 3:**
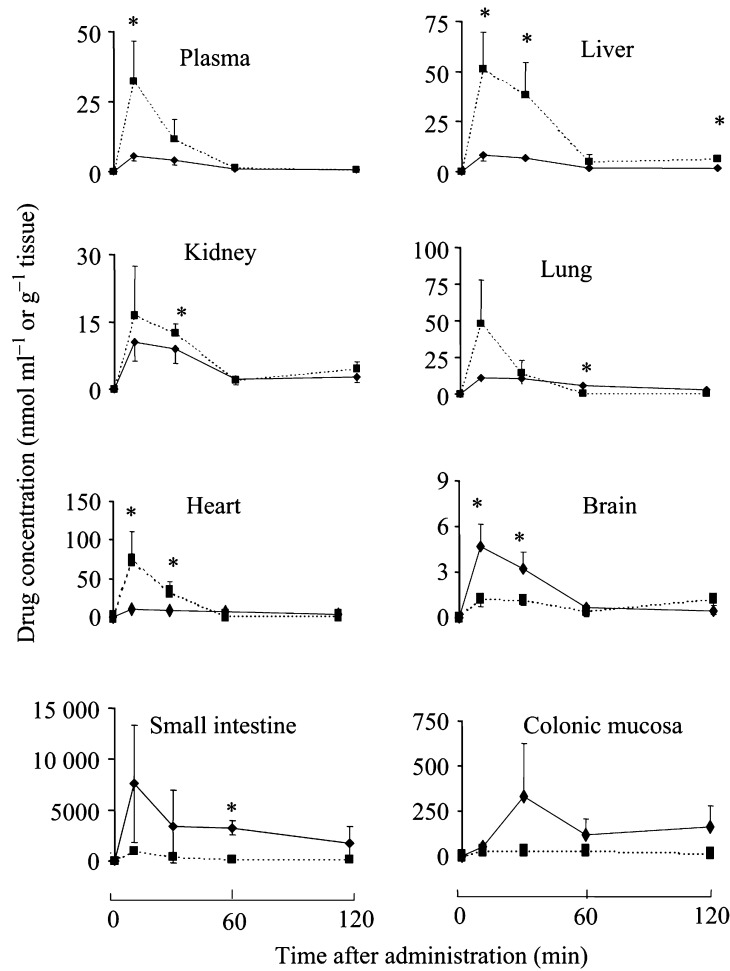
Concentrations of resveratrol (squares, dotted line) and DMU 212 (rhombi, solid line) in the plasma and tissues of mice that received a single dose of drug (240 mg kg^−1^) i.g. Values are the mean±s.d. (*n*=3). Star indicates that the values differ significantly (*P*<0.05, one-way ANOVA). For details of dosing, extraction and HPLC analyses, see Materials and Methods.

Mice received intragastric resveratrol or DMU 212 (1 or 0.8 mmol kg^−1^), and drug levels were measured in the plasma and liver, kidney, lung, brain, small intestinal mucosa and colonic mucosa. These are all tissues in which resveratrol might prevent malignancy, or delay its onset. For comparison, levels in the heart were also studied. The results are shown in Figure 3

. The plasma and tissue level data were used to calculate the respective mean AUC_0–120 min_ values (Table 1

**Table 1:** Area under the plasma or tissue concentration time curve (AUC) for resveratrol and DMU 212 in mice that received these agents (240mg/kg) i.g

	**AUC (nmoles ml^−1^ or g^−1^ min)**		
**Tissue**	**Resveratrol**	**DMU 212**	**AUC_res_/AUC_DMU_**
Plasma	863	246	3.5
Liver	2150	432	5
Kidney	785	566	1.5
Lung	1123	778	1.5
Heart	2072	750	3
Brain	103	193	0.5
Intestinal mucosa	36 690	369 315	0.1
Colonic mucosa	2869	19 256	0.15

AUC values were calculated from the curves shown in Figure 2 using the mean plasma or tissue concentration values between 0 and 120 min *post*administration.

). Both agents were rapidly cleared from blood and tissues within an hour of administration (Figure 3). Concentrations of resveratrol were consistently higher than those of DMU 212 in the plasma, liver and heart. Levels were similar in the kidney and lung, while resveratrol concentrations were much below those of DMU 212 in the brain, small intestinal and colonic mucosae. The most dramatic discrepancy in levels occurred in the liver, in which the AUC for resveratrol was five times higher than that for DMU 212, and in the small intestinal and colonic mucosae, in which the AUCs for DMU 212 exceeded those for resveratrol by factors of 10 and 7, respectively (Table 1). Resveratrol levels peaked after 10 min, the first of the time points chosen, and peak concentrations were 32 *μ*M in plasma and 51, 16, 50, 1.2, 75, 960 and 30 nmols g^−1^ tissue in the liver, kidney, lung, brain, heart, small intestinal and colonic mucosae, respectively. DMU 212 levels reached a peak after 10 min, except in the case of the colon, in which the peak occurred after 30 min. The peak levels for DMU 212 were 5 *μ*M in the plasma and 8, 11, 11, 5, 10, 7600 and 330 nmols g^−1^ tissue in the liver, kidney, lung, brain, heart, small intestinal and colonic mucosae, respectively. We performed a preliminary experiment in order to explore whether the difference in gastrointestinal levels would also be observed when the agents are administered with the diet, the route often used in preclinical cancer chemoprevention studies. Agents were admixed with the diet at 0.2% and fed for 2 weeks. Colonic levels of DMU 212 after DMU 212 administration exceeded those of resveratrol after resveratrol administration by two-fold (result not shown).

### Metabolism of resveratrol and DMU 212

**Figure 4:**
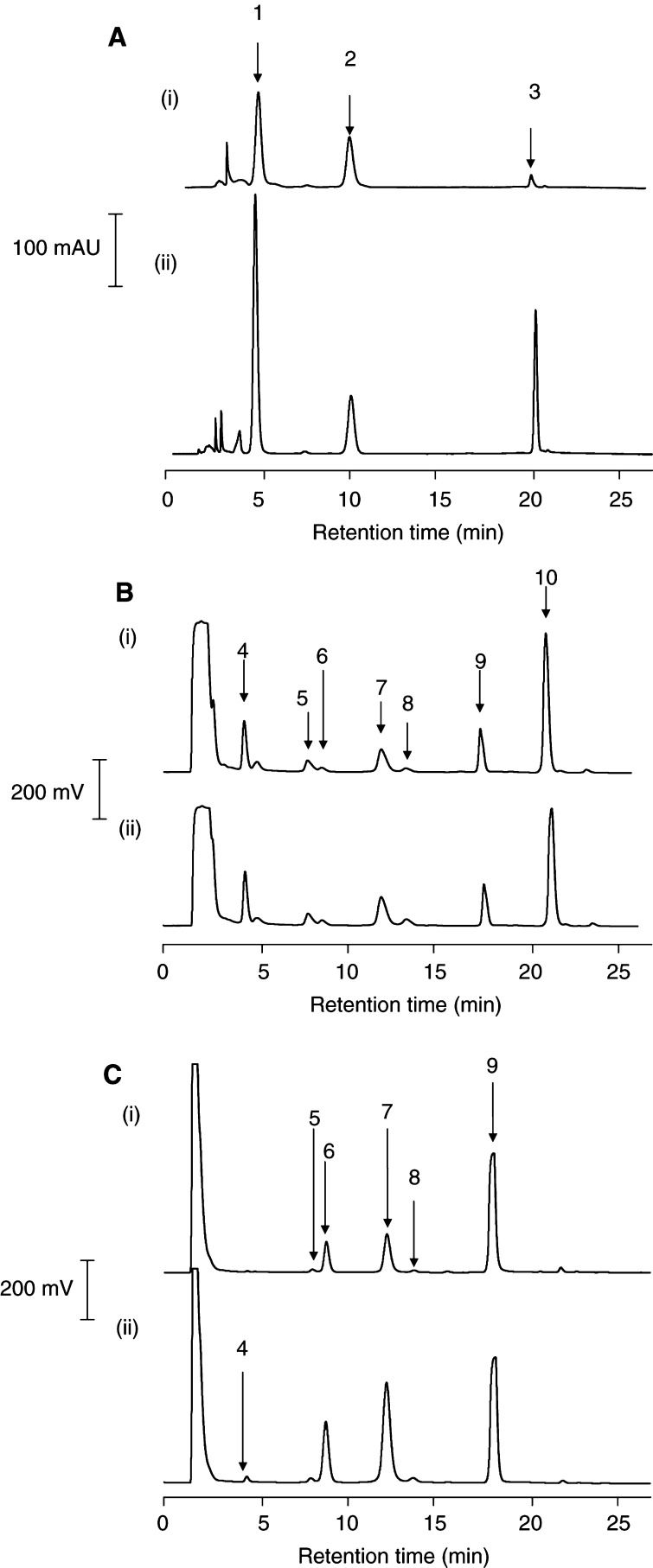
(**A**) HPLC analysis of liver extracts of mice that received resveratrol (240 mg kg^−1^) p.o. (i) and of a mixture (ii) of (i) and biosynthesised resveratrol glucuronide. Liver tissue was obtained 60 min postadministration. Peak allocation is (1) resveratrol glucuronide, (2) resveratrol sulphate and (3) resveratrol. The chromatogram is representative of three. For details of dosing, extraction and HPLC analyses, see Materials and Methods. (**B**) HPLC analysis of liver extracts of mice that received DMU 212 (240 mg kg^−1^) p.o. (i) a mixture (ii) of (i) with authentic standards DMU 212, 4,4′-di-desmethyl-DMU 212 (DMU 295), 4′-desmethyl-DMU 212 (DMU 281), 3′-hydroxy-DMU 212 (DMU 214), 4-desmethyl-DMU 212 (DMU 291) and 3-desmethyl-DMU 212 (DMU 807). Liver tissue was obtained 60 min postadministration. Peak allocation is (4) 4,4′-di -desmethyl-DMU 212 (DMU 295) (5) 4′-desmethyl-DMU 212 (DMU 281), (6) 3′-hydroxy-DMU 212 (DMU 214), (7) 4-desmethyl-DMU 212 (DMU 291), (8) 3-desmethyl-DMU 212 (DMU 807) (9) DMU 212 and (10) internal standard. The chromatogram is representative of three. For details of dosing, extraction and HPLC analyses, see Materials and Methods. (**C**) HPLC analysis of extracts of an incubate of mouse liver microsomes with DMU 212 (1 mM) (i) and of a mixture (ii) of (i) with authentic DMU 212, 4,4′-di-desmethyl-DMU 212 (DMU 295), 4′-desmethyl-DMU 212 (DMU 281), 3′-hydroxy-DMU 212 (DMU 214), 4-desmethyl-DMU 212 (DMU 291) and 3-desmethyl-DMU 212 (DMU 807). Incubations were terminated after 20 min. Peak allocation is (4) 4,4′-di-desmethyl-DMU 212 (DMU 295) (5) 4′-desmethyl-DMU 212 (DMU 281), (6) 3′-hydroxy-DMU 212 (DMU 214), (7) 4-desmethyl-DMU 212 (DMU 291), (8) 3-desmethyl-DMU 212 (DMU 807) (9) DMU 212. The chromatogram is representative of three. For details of incubation, extraction and HPLC analysis, see Materials and Methods.

Plasma and tissues of mice, which had received resveratrol or DMU 212, were investigated using HPLC cochromatography and mass spectrometry in order to detect and characterise metabolites. HPLC analysis of liver samples from animals on resveratrol displayed two peaks (Figure 4A

) in addition to the parent compound. These two species were also observed in the liver, lung and kidney, but not in the plasma (result not shown). Solvent eluting from the column with the two peaks was collected, and peak constituents were isolated and subjected to mass spectrometric analysis. Mass spectrometric analysis of peak 1 afforded the molecular ion *m/z*=403, consistent with a resveratrol glucuronide. Biosynthesis of resveratrol glucuronide using UDP glucuronyl transferase and liver microsomes generated material, which on cochromatography eluted with peak 1 (Figure 4A). Mass spectral analysis did not allow unequivocal confirmation of its structural assignment as either the 3 or 4′ glucuronide. Peak 2 eluted with the same retention time as authentic resveratrol-3-sulphate and yielded *m/z*=307, consistent with resveratrol sulphate.

Extracts of plasma and liver samples from animals that had received DMU 212 exhibited several peaks in addition to that of the parent drug (Figure 4B). Extracts were subjected to cochromatography with several authentic putative metabolic products of mono-hydroxylation or *O*-demethylation of DMU 212. The results suggest that five of the peaks were 4,4′-dihydroxy-3,5-dimethoxystilbene (DMU 295, 4,4′-di-desmethyl-DMU 212, for structures see Figure 2), 4′-hydroxy-3,4,5-trimethoxystilbene (DMU 281, 4′-desmethyl-DMU 212), 3′-hydroxy-3,4,5,4′-tetramethoxystilbene (DMU 214, 3′-hydroxy-DMU 212), 4-hydroxy-3,5,4′-trimethoxystilbene (DMU 291, 4-desmethyl-DMU 212), and 3-hydroxy-4,5,4′-trimethoxystilbene (DMU 807, 3-desmethyl-DMU 212). Collection of solvent eluting individual peaks and subsequent tandem mass spectrometric analysis of solvent extracts confirmed the putative structural assignment for the four metabolites 4,4′-di-desmethyl-DMU 212 (*m/z*=273, Table 2

**Table 2:** Features of daughter ion spectra obtained by tandem mass spectrometry of molecular ions (M+H)^+^ of DMU 212 and four metabolites isolated from peaks in high-performance liquid chromatograms of extracts of livers of micethat had received DMU 212 (240 mg kg^−1^) and of peaks of authentic reference compounds

	**Authentic standard *m/z***	**Liver extract *m/z***
DMU 212	301 (M+H)^+^ (55)	301 (M+H)^+^ (80)
	270 (100)	270 (100)
	286 (35)	286 (50)
		
4,4′-Di-desmethyl-DMU 212	273 (M+H)^+^ (23)	273 (M+H)^+^ (25)
	198 (100)	198 (50)
	181 (62)	181 (81)
		
3-Desmethyl-DMU 212	287 (M+H)^+^ (10)	287 (M+H)^+^ (75)
	227 (72)	227 (20)
	195 (70)	195 (18)
		
4′-Desmethyl-DMU 212	287 (M+H)^+^ (10)	287 (M+H)^+^ (60)
	256 (100)	256 (100)
	272 (80)	272 (47)
		
3′-Hydroxy-DMU 212	317 (M+H)^+^ (31)	317 (M+H)^+^ (21)
	225 (55)	225 (100)
	286 (22)	286 (10)

Only three prominent product ions are shown. Relative abundance in percentage is shown in parentheses.

), 4′-desmethyl-DMU 212 (*m/z*=287), 3′-hydroxy-DMU 212 (*m/z*=317) and 3-desmethyl-DMU 212 (*m/z*=287). In extracts of lung and kidney tissues, these metabolites were also detected, albeit at lower concentrations (result not shown).

For confirmatory purposes, the metabolism of resveratrol and DMU 212 was also studied *in vitro* in suspensions of liver microsomes fortified with cofactors of cytochrome *P*450 enzymes. While HPLC analysis of extracts of the incubation mixture with resveratrol did not yield any peak in addition to that of the parent substrate, analysis of incubates with DMU 212 afforded peaks in addition to that of parent DMU 212 (Figure 4C). In analogy to the analysis of liver tissue from mice that had received DMU 212, the incubation mixture was extracted and the extract was subjected to cochromatographic analysis. The results (Figure 4C) suggest that 4,4′-di-desmethyl-DMU 212, 4′-desmethyl-DMU 212, 3′-hydroxy-DMU 212, 4-desmethyl-DMU 212 and 3-desmethyl-DMU 212 were products of the microsomal biotransformation of DMU 212.

### Effect of resveratrol and DMU 212 on the growth of colon cancer cells

**Figure 5:**
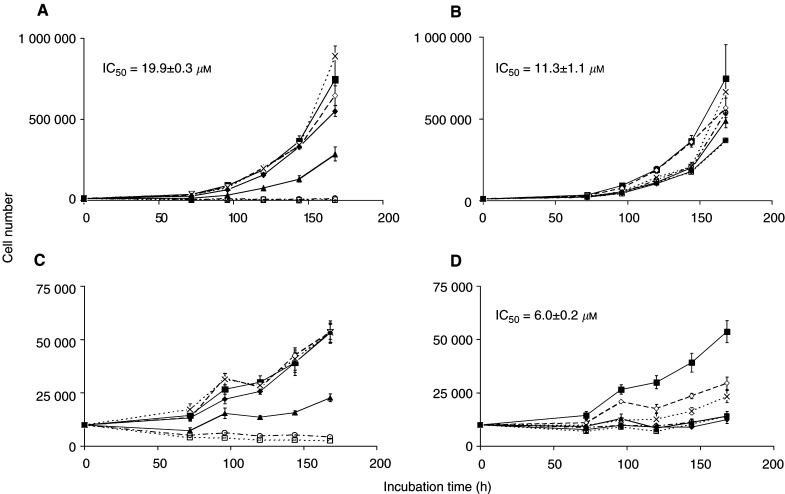
Effect of resveratrol (**A**, **C**) and DMU 212 (**B**, **D**) on the growth of HT-29 (**A**, **B**) and HCA-7 colon cancer cells (**C**, **D**). Symbols indicate the following agent concentrations: closed squares control cells, open rhombi 1 *μ*M, crosses 5 *μ*M, closed rhombi 10 *μ*M, closed triangles 25 *μ*M, open circles 50 *μ*M and open squares 100 *μ*M. IC_50_ values computed for the 168 h time point are inserted. Values are the mean±s.d. of four independent experiments.

The growth-modulating ability of resveratrol and DMU 212 were investigated in a preliminary manner in HT-29 and HCA-7 colon cancer cells *in vitro*. The IC_50_ values computed form the growth curves (Figure 5

) range from 6 to 26 *μ*M, and DMU 212 appears to be a slightly more potent growth inhibitor than resveratrol.

## DISCUSSION

The results presented above suggest that the introduction of four methoxy groups into the stilbene framework, three of which replaced the hydroxy moieties in resveratrol, fails to increase the systemic availability of the molecule in comparison to resveratrol. This conclusion is borne out by the comparison between resveratrol and DMU 212 in terms of the concentrations of these agents in the plasma, liver and heart after oral administration, in which the availability of DMU 212 was inferior to that of resveratrol. In contrast, in comparison to resveratrol, DMU 212 was found to be more available in intestinal and colonic mucosae and in the brain. *A priori*, it is difficult to predict on theoretical grounds in what manner such a structural modification might alter the pharmacokinetic profile of the stilbene molecule. One of the physicochemical corollaries of this alteration, which undoubtedly impacts on the pharmacokinetics of the molecule, is the increase in lipophilicity conferred on the stilbene species in DMU 212 by four methoxy functionalities *vis*-*á*-*vis* resveratrol, which has three hydroxy groups instead. The difference in lipophilicity between DMU 212 and resveratrol is reflected by the fact that reversed phase HPLC analysis of a mixture of both agents using the gradient system described under Materials and Methods for DMU 212 afforded a retention time of 17 min for DMU 212, while resveratrol eluted with the solvent front.

Another factor, which undoubtedly determines the differential pharmacokinetic properties of resveratrol and DMU 212, is the divergence in their metabolic profile. Resveratrol is known to undergo metabolic phase II reactions involving conjugation with sulphate and glucuronic acid (Yu *et al*, 2002). Consistent with this report, we found a resveratrol sulphate and a resveratrol glucuronide in the liver and other tissues of mice that had received resveratrol. In contrast to resveratrol, DMU 212 was subjected to hepatic metabolic oxidation, especially single or double *O*-demethylation reactions in the 3, 4 or 4′ positions of the molecule. In addition, we could identify a hydroxylated metabolic species (3′-hydroxy-DMU 212, DMU 214). The spectrum of *O*-demethylated and hydroxylated metabolites found *in vivo* was accurately reflected by the metabolic profile obtained on incubation of DMU 212 with NADPH-fortified liver microsomes. These findings are consistent with the results of recent *in vitro* experiments using cytochrome *P*450 isoenzyme preparations, in which DMU 212 was found to undergo both aromatic hydroxylation and *O*-demethylation reactions primarily catalysed by isoenzymes of the CYP1 family (Wilsher *et al*, unpublished). In analogy, resveratrol was recently found to undergo metabolic oxidation *in vitro* to piceatannol (3,5,2′,4′-tetra-hydroxystilbene), when incubated with a source of CYP1B1 (Potter *et al*, 2002b). In the study described here, piceatannol was not identified as a metabolite of resveratrol in mice *in vivo* or in mouse liver microsomes *in vitro*. It is conceivable that piceatannol was present at very low levels, which might have confounded detection by the procedures used here. The ability to convert resveratrol to piceatannol has been surmised to be a relatively specific property of CYP1B1 (Potter *et al*, 2002b). The lack of the presence of piceatannol at detectable concentrations in our study in the mouse is consistent with the notion that CYP1B1 is not expressed at appreciable levels in mouse liver (Shimada *et al*, 2003). Overall, the result described here suggests that hydroxylation to piceatannol is probably not a major metabolic route for resveratrol in the mouse.

It is conceivable that 4,4′-di-desmethyl-DMU 212, 4′-desmethyl-DMU 212, 3′-hydroxy-DMU 212 and 3-desmethyl-DMU 212, the DMU 212 metabolites identified here, undergo phase II metabolism to yield sulphate and/or glucuronide metabolites. However, conjugates of metabolic species derived from DMU 212 were not found in the mouse. We cannot exclude that such conjugates were formed *in vivo* but not detected. Nevertheless, there were no peaks indicative of such conjugates, characterised by short retention times, which could have been subjected to mass spectral investigation. It has to be stressed that the metabolism studies described here did not allow quantification of the metabolic species identified, so that it is not possible to adjudge the difference between resveratrol and DMU 212 in terms of the contribution of hepatic metabolism to the overall systemic clearance of the molecules.

The pharmacokinetic properties of DMU 212 have thus far been unknown. In contrast, resveratrol has been the subject of several pharmacokinetic studies, including those in the rat (Soleas *et al*, 2001) and the mouse (Vitrac *et al*, 2003), in which resveratrol has been administered via the oral route. In terms of peak levels and systemic disappearance, the results outlined above in mice are consistent with the published studies. The increased drug levels in the liver, kidney, lung and heart obtained after ingestion of resveratrol in comparison to those after DMU 212 reflect the difference in availability observed in the plasma. In contrast, the levels of DMU 212 in the brain, small intestinal and colonic mucosae after DMU 212 administration exceeded levels of resveratrol measured after resveratrol intake. The higher availability of DMU 212 in the brain suggests that it is capable of crossing the blood–brain barrier more easily than resveratrol, which is probably a consequence of the higher lipophilicity of DMU 212. The discrepancy between resveratrol and DMU 212 in concentration achieved in the small intestine and colon may be a corollary of their differential metabolic susceptibilities. The lower availability of resveratrol compared to DMU 212 may be the consequence of the high propensity of resveratrol to undergo conjugation reactions catalysed by enzymes (De Santi *et al*, 2000; Kuhnle *et al*, 2000; Marier *et al*, 2002), which are abundantly present in the gut (Eisenhofer *et al*, 1999). In contrast, on the basis of the fact that activities of oxidising enzymes in the gut are much lower than in the liver (Doherty and Charman, 2002), the ability of the gut mucosa to *O*-demethylate or hydroxylate DMU 212 is arguably much lower than its ability to biotransform resveratrol by conjugation. On the speculative assumption that DMU 212 and resveratrol share biochemical mechanisms germane to cancer chemoprevention and that DMU 212 is not inferior to resveratrol in intrinsic potency, this observation hints at a potential advantage of DMU 212 over resveratrol when applied as an experimental colorectal cancer chemopreventive agent. The superior growth-inhibitory and apoptotic potency of DMU 212 in comparison to resveratrol in transformed human lung-derived cells (Lu *et al*, 2001) is consistent with this hypothesis. Peak levels of DMU 212 that were achieved in the colonic and small intestinal mucosae in mice exceeded those required to cause significant arrest of transformed lung cell growth *in vitro* (10 *μ*M) by factors of 32 and 760, respectively. Furthermore, the results of our preliminary assessment of the colon cancer cell growth-inhibitory properties of resveratrol and DMU 212 suggest that the concentrations of either agent required to inhibit growth, with IC_50_ values between 6 and 26 *μ*M, are comfortably within the range achieved in the colorectal tract of mice that received these agents at the oral doses used in the study described here, 240 mg kg^−1^.

In the light of the suggestion that resveratrol at 0.01% in the drinking water (constituting a dose of ∼15 mg kg^−1^ per day) decreased adenoma multiplicity in the Apc^*Min*/+^ mouse model (Schneider *et al*, 2001), the results described here render a comparison of resveratrol and DMU 212 in this model appropriate, and such experiments are currently planned in this laboratory. However, it is important to add a note of caution, because the chemopreventive efficacy of resveratrol in this model is highly contentious, as borne out by two contradictory abstracts published subsequent to the paper by Schneider *et al* (2001). These abstracts suggest that in the same murine model, dietary doses comparable to, or much higher than, those used by Schneider *et al* were completely ineffective (Ziegler *et al*, 2001) or, in the case of a dietary daily dose of 500 mg kg^−1^ for 14 days, reduced adenoma load, but did so only in female mice and not at all in male mice (Gignac and Bourquin, 2001).

In conclusion, the work described here provides an initial pharmacokinetic groundwork, which can contribute to rational decision making as to the choice of resveratrol analogues that should be selected for comparative testing for cancer chemopreventive potency in preclinical models. DMU 212 showed more favourable pharmacokinetic properties than resveratrol, in that it yielded higher levels of drug in the small intestinal, colonic mucosae and brain. Buttressed by our recent finding that DMU 212 is devoid of any toxicity in rats when administered at single doses of up to 40 mg kg^−1^ via the i.v. route or up to 400 mg kg^−1^ when administered p.o. (Verschoyle *et al*, unpublished), the results presented here render the exploration of DMU 212 side by side with resveratrol for chemopreventive efficacy in rodent models of colorectal carcinogenesis propitious.

## References

[bib1] Asensi M, Medina I, Ortega A, Carrertero J, Bano MC, Obrador E, Estrela JM (2002) Inhibition of cancer growth by resveratrol is related to its low bioavailability. Free Radical Biol Med 33: 387–398 12126761 10.1016/s0891-5849(02)00911-5

[bib2] Banerjee S, Bueso-Ramos C, Aggarwal BB (2002) Suppression of 7,12 dimethylbenz(*a*)anthracene-induced mammary carcinogenesis in rats by resveratrol: role of nuclear factor-kappa B, cyclooxygenase 2, and matrix metalloprotease 9. Cancer Res 62: 4945–4954 12208745

[bib3] Bertelli AAE, Giovannini R, Stradi R, Urien S, Tillement JP, Bertelli A (1996) Kinetics of trans- and cis-resveratrol (3,4′,5-trihydroxystilbene) after red wine oral administration in rats. Int J Clin Pharm Res 16: 77–81 9172004

[bib4] Bhat KPL, Lantvit D, Christov K, Mehta RG, Moon RC, Pezzuto JM (2001) Estrogenic and antiestrogenic properties of resveratrol in mammary tumor models. Cancer Res 61: 7456–7463 11606380

[bib5] Chun YJ, Kim MY, Guengerich FP (1999) Resveratrol is a selective human cytochrome *P*450 1A1 inhibitor. Biochem Biophys Res Commun 262: 20–24 10448061 10.1006/bbrc.1999.1152

[bib6] Chun YJ, Kim S, Kim D, Lee SK, Guengerich FP (2001) A new selective and potent inhibitor of human cytochrome *P*450 1B1 and its application to antimutagenesis. Cancer Res 61: 8164–8170 11719446

[bib7] De Santi C, Pietrabissa A, Mosca F, Pacifici GM (2000) Glucuronidation of resveratrol, a natural product present in grape and wine, in the human liver. Xenobiotica 30: 1047–1054 11197066 10.1080/00498250010002487

[bib8] Doherty MM, Charman WN (2002) The mucosa of the small intestine: how clinically relevant as an organ of drug metabolism? Clin Pharmacokinet 41: 235–253 11978143 10.2165/00003088-200241040-00001

[bib9] Eisenhofer G, Coughtrie MW, Goldstein DS (1999) Dopamine sulphate: an enigma resolved. Clin Exp Pharmacol Physiol 26: S41–S53 10386253

[bib10] Gehm BD, McAndrews JM, Chien PY, Jameson JL (1997) Resveratrol, a polyphenolic compound found in grapes and wine, is an agonist for the estrogen receptor. Proc Natl Acad Sci USA 94: 14138–14143 9391166 10.1073/pnas.94.25.14138 PMC28446

[bib11] Gignac EA, Bourquin LD (2001) Influence of resveratrol and sulindac on intestinal tumor numbers in Min mice. FASEB J 15: A630

[bib12] Goldberg DA, Yan J, Soleas GJ (2003) Absorption of three wine-related polyphenols in three different matrices by healthy subjects. Clin Biochem 36: 79–87 12554065 10.1016/s0009-9120(02)00397-1

[bib13] Guengerich FP, Chun YJ, Kim D, Gillam EMJ, Shimada T (2003) Cytochrome *P*4501B1: a target for inhibition in anticarcinogenesis strategies. Mutat Res 523–524: 173–182 10.1016/s0027-5107(02)00333-0 12628515

[bib14] Gusman J, Malonne H, Atassi G (2001) A re-appraisal of the potential chemopreventive and chemotherapeutic properties of resveratrol. Carcinogenesis 22: 1111–1117 11470738 10.1093/carcin/22.8.1111

[bib15] Haworth RS, Avkiran M (2001) Inhibition of protein kinase D by resveratrol. Biochem Pharmacol 62: 1647–1651 11755118 10.1016/s0006-2952(01)00807-3

[bib16] Jang MS, Cai EN, Udeani GO, Slowing KV, Thomas CF, Beecher CWW, Fong HHS, Farnsworth NR, Kinghorn DA, Mehta RG, Moon RC, Pezzuto JM (1997) Cancer chemopreventive activity of resveratrol, a natural product derived from grapes. Science 275: 218–220 8985016 10.1126/science.275.5297.218

[bib17] Juan ME, Buenafuente J, Casals I, Planas JM (2002) Plasmatic levels of *trans*-resveratrol in rats. Food Res Int 35: 195–199

[bib18] Kim S, Ko H, Park JE, Jung S, Lee SK, Chun YJ (2002) Design, synthesis, and discovery of novel *trans*-stilbene analogues as potent and selective human cytochrome *P*4501B1 inhibitors. J Med Chem 45: 160–164 11754588 10.1021/jm010298j

[bib19] Kuhnle G, Spencer JPE, Chowrimootoo G, Schroeter H, Debnam ES, Srai SKS, Rice Evans C, Hahn U (2000) Resveratrol is absorbed in the small intestine as resveratrol glucuronide. Biochem Biophys Res Commun 272: 212–217 10872829 10.1006/bbrc.2000.2750

[bib20] Li ZG, Hong T, Shimada Y, Komoto I, Kawabe A, Ding Y, Kaganoi J, Hashimoto Y, Imamura M (2002) Suppression of *N*-nitrosomethylbenzylamine (NMBA)-induced esophageal tumorigenesis in F344 rats by resveratrol. Carcinogenesis 23: 1531–1536 12189197 10.1093/carcin/23.9.1531

[bib21] Lu JB, Ho CT, Ghai G, Chen KY (2001) Resveratrol analog, 3,4,5,4′-tetrahydroxystilbene, differentially induces pro-apoptotic p53/Bax gene expression and inhibits the growth of transformed cells but not their normal counterparts. Carcinogenesis 22: 321–328 11181455 10.1093/carcin/22.2.321

[bib22] Mahyar-Roemer M, Katsen A, Mestres P, Roemer K (2001) Resveratrol induces colon tumor cell apoptosis independently of p53 and preceded by epithelial differentiation, mitochondrial proliferation and membrane potential collapse. Int J Cancer 94: 615–622 11745454 10.1002/ijc.1516

[bib23] Mahyar-Roemer M, Kohler H, Roemer K (2002) Role of Bax in resveratrol-induced apoptosis of colorectal carcinoma cells. BMC Cancer 2: 27–35 12383351 10.1186/1471-2407-2-27 PMC130964

[bib24] Marier JF, Vachon P, Gritsas A, Zhang J, Moreau J-P, Ducharme MP (2002) Metabolism and disposition of resveratol in rats: extent of absorption, glucuronidation, and enterohepatic recirculation evidenced by a linked-rat model. J Pharm Exp Ther 302: 369–373 10.1124/jpet.102.033340 12065739

[bib25] Mutoh M, Takahashi M, Fukuda K, Matsushima-Hibiya Y, Mutoh H, Sugimura T, Wakabayashi K (2000) Suppression of cyclooxygenase-2 promoter-dependent transcriptional activity in colon cancer cells by chemopreventive agents with a resorcin-type structure. Carcinogenesis 21: 959–963 10783318 10.1093/carcin/21.5.959

[bib26] Nam KA, Kim S, Heo YH, Lee SK (2001) Resveratrol analog 3,5,2′,4′-tetramethoxy-*trans*-stilbene, potentiates the inhibition of cell growth and induces apoptosis in human cancer cells. Arch Pharmacol Res 24: 441–445 10.1007/BF02975192 11693548

[bib27] Pettit GR, Singh SB, Boyd MR, Hamel E, Pettit RK, Schmidt JM, Hogan F (1995) Antineoplastic agents 291. Isolation and synthesis of combretastatin A4, A5 and A6. J Med Chem 38: 1666–1672 7752190 10.1021/jm00010a011

[bib28] Potter GA, Butler PC, Ruparelia KC, Ijaz T, Wilsher NC, Wanogho E, Tan HL, Hoang TTV, Stanley LA, Burke MD (2002a) DMU212: a novel CYP1B1 activated anticancer prodrug. Br J Cancer 86(Suppl 1): S117

[bib29] Potter GA, Patterson LH, Burke MD, Butler PC (1999) Aromatic hydroxylation activated prodrugs. Int Patent Applic No. WO 99/40056

[bib30] Potter GA, Patterson LH, Wanogho E, Perry PJ, Butler PC, Ijaz T, Ruparelia KC, Lamb JH, Farmer PB, Stanley LA, Burke MD (2002b) The cancer preventative agent resveratrol is converted to the anticancer agent piceatannol by the cytochrome *P*450 enzyme CYP1B1. Br J Cancer 86: 774–778 11875742 10.1038/sj.bjc.6600197 PMC2375304

[bib31] Schneider Y, Duranton B, Gosse F, Schleiffer R, Seiler N, Raul F (2001) Resveratrol inhibits intestinal tumorigenesis and modulates host-defense-related gene expression in an animal model of human familial adenomatous polyposis. Nutr Cancer 39: 102–107 11588890 10.1207/S15327914nc391_14

[bib32] Schneider Y, Vincent F, Duranton B, Badolo L, Gosse F, Bergmann C, Seiler N, Raul F (2000) Anti-proliferative effect of resveratrol, a natural component of grapes and wine, on human colonic cancer cells. Cancer Lett 158: 85–91 10940513 10.1016/s0304-3835(00)00511-5

[bib33] Sgambato A, Ardito R, Faraglia B, Boninsegna A, Wolf FI, Cittadini A (2001) Resveratrol, a natural phenolc compound, inhibits cell proliferation and prevents oxidative DNA damage. Mutat Res Gen Toxicol Env Mutagen 496: SI171–SI180 10.1016/s1383-5718(01)00232-7 11551493

[bib34] She QB, Ma WY, Wang MF, Kaji A, Ho CT, Dong ZG (2003) Inhibition of cell transformation by resveratrol and its derivatives: differential effects and mechanisms involved. Oncogene 22: 2143–2150 12687016 10.1038/sj.onc.1206370

[bib35] Shimada T, Sugie A, Shindo M, Nakajima T, Azuma E, Hashimoto M, Inoue K (2003) Tissue-specific induction of cytochrome *P*450 1A1 and 1B1 by polycyclic aromatic hydrocarbons and polychlorinated biphenyls in engineered C57BL/6J mice of arylhydrocarbon receptor gene. Toxicol Appl Pharmacol 187: 1–10 12628579 10.1016/s0041-008x(02)00035-2

[bib36] Slater SJ, Seiz JL, Cook AC, Stagliano BA, Buzas CJ (2003) Inhibition of protein kinase C by resveratrol. Biochem Biophys Acta 1637: 59–69 12527408 10.1016/s0925-4439(02)00214-4

[bib37] Soleas GJ, Angelini M, Grass L, Diamandis EP, Goldberg DM (2001) Absorption of *trans*-resveratrol in rats. Methods Enzymol 335: 145–154 11400363 10.1016/s0076-6879(01)35239-4

[bib38] Subbaramaiah K, Chung WJ, Michaluart P, Telang N, Tanabe T, Inoue H, Jang MS, Pezzuto JM, Dannenberg AJ (1998) Resveratrol inhibits cyclooxygenase-2 transcription and activity in phorbol ester-treated human mammary epithelial cells. J Biol Chem 273: 21875–21882 9705326 10.1074/jbc.273.34.21875

[bib39] Surh YJ, Chun KS, Cha HH, Han SS, Keum YS, Park KK, Lee SS (2001) Molecular mechanisms underlying chemopreventive activities of anti-inflammatory phytochemicals: down-regulation of COX-2 and iNOS through suppression of NF-kB activation. Mutat Res 480–481: 243–268 10.1016/s0027-5107(01)00183-x 11506818

[bib40] Tessitore L, Davit A, Sarotto I, Caderni G (2000) Resveratrol depresses the growth of colorectal aberrant crypt foci by affecting bax and p21(CIP) expression. Carcinogenesis 21: 1619–1622 10910967

[bib41] Uenobe F, Nakamura S, Miyazawa M (1997) Antimutagenic effect of resveratrol against Trp-P-1. Mutat Res 373: 197–200 9042400 10.1016/s0027-5107(96)00191-1

[bib42] Vitrac X, Desmouliere A, Brouillaud B, Krisa S, Deffieux G, Barthe N, Rosenbaum J, Merillon JM (2003) Distribution of [14C]-trans-resveratrol, a cancer chemopreventive polyphenol, in mouse tissues after oral administration. Life Sci 72: 2219–2233 12628442 10.1016/s0024-3205(03)00096-1

[bib43] Wolter F, Akoglu B, Clausnitzer A, Stein J (2001) Downregulation of the cyclin D1/Cdk4 complex occurs during resveratrol-induced cell cycle arrest in colon cancer cell lines. J Nutr 131: 2197–2203 11481417 10.1093/jn/131.8.2197

[bib44] Yu CW, Shin YG, Chow A, Li YM, Kosmeder JW, Lee YS, Hirschelman WH, Pezzuto JM, Mehta RG, van Breeman RB (2002) Human, rat, and mouse metabolism of resveratrol. Pharm Res 19: 1907–1914 12523673 10.1023/a:1021414129280

[bib45] Ziegler CC, McEntree MF, Hansen-Petrik M, Johnson BT, Whelan J (2001) *In vivo* effect of *trans*-resveratrol on intestinal tumorigenesis. FASEB J 15: A617

